# Algal Biodiversity of Nine Megaliths in South-East Bulgaria

**DOI:** 10.3390/life14080948

**Published:** 2024-07-28

**Authors:** Maya Stoyneva-Gärtner, Miroslav Androv, Blagoy Uzunov, Kristian Ivanov, Georg Gärtner

**Affiliations:** 1 Department of Botany, Faculty of Biology, Sofia University “St. Kliment Ohridski”, 8 blvd. Dragan Tsankov, 1164 Sofia, Bulgaria; mstoyneva@uni-sofia.bg (M.S.-G.); androv@uni-sofia.bg (M.A.); krisanri@uni-sofia.bg (K.I.); 2 Institute of Botany, Innsbruck University, Sternwartestrasse 15, 6020 Innsbruck, Austria; georg.gaertner@uibk.ac.at

**Keywords:** aeroterrestrial algae, Bacillariophyceae, Chlorophyta, Cyanobacteria, Cyanoprokaryota, drone, Eustigmatophyceae, Ochrophyta, Streptophyta, Xanthophyceae

## Abstract

This paper presents the first data on the biodiversity of lithophytic algae from Bulgarian megaliths obtained after the application of the direct sampling method, subsequent cultivation, and processing by light microscopy. A rich algal flora was found: 90 species and 1 variety of 65 genera from Cyanoprokaryota/Cyanobacteria (29 species, 13 genera), Chlorophyta (40 species and 1 variety, 38 genera), Streptophyta (5 species, 1 genus), and Ochrophyta (16 species, 13 genera). Among them were the globally rare *Pseudodictyochloris multinucleata* (Chlorophyta), found for the first time in such lowland and warm habitats, and *Scotiella tuberculata* (Chlorophyta), for which this is the first finding in the country. Three of the recorded species are conservationally important. The low floristic similarity between the sites (0–33%) shows the diversity of the algal flora, with no common species found for all the megaliths studied. The most widespread were the strongly adaptive and competitive *Stichococcus bacillaris*, *Apatococcus lobatus*, and *Chloroidium ellipsoidium* (Chlorophyta). The correlations estimated between the species number and substrate temperature (18.1–49.6 °C) suggest the prospect of future research related to the impact of global warming. In addition, the study points to the safety aspects as it revealed species from nine potentially toxin-producing cyanoprokaryotic genera that could be harmful to visitors’ health.

## 1. Introduction

Currently, problems affecting aquatic systems and organisms related to rapidly advancing climate changes and eutrophication are recognized worldwide, and scientific publications on these topics appear in emerging ways. However, much less attention is paid to the effects of both expanded eutrophication of the atmosphere (caused by increased nitrogen compounds and hydrocarbons) and of global warming on the land, aeroterrestrial environments, and their inhabitants [1]. More than 1000 algae, both prokaryotic and eukaryotic, form an important part of the rich aeroterrestrial microbial life on hard natural substrates such as soils, rocks, stones, tree barks, and different manufactured surfaces, including fences, roofs, building facades, and monuments [1,2,3]. However, they are much less studied compared to their aquatic counterparts [1,4] mainly because their reliable identification requires time-consuming and labor-intensive cultivation-dependent methods [5,6]. Nevertheless, interest in these organisms has been increasing over the years for several reasons: (1) they serve as an essential organic-matter basis for heterotrophic organisms due to their photosynthetic ability and their role as primary producers [7]; (2) living in outdoor habitats, they are directly exposed to all adverse environmental factors and include some rare and threatened species (e.g., [8]); (3) there is currently increasing evidence that is useful for modern biotechnology, food industry, medicine, pharmacy, cosmetics, etc. [4,9,10]; (4) some of them are able to synthesize toxic substances that are harmful for ecosystem and human health [11,12]; (5) there is a currently growing interest to non-suspended, attached cultivation that offers higher biomass yields, better control of contamination, and lower water consumption compared to ordinary suspended photobioreactors [13,14]; (6) in the long term, algae, together with all other inhabitants of hard substrates (e.g., bacteria, mosses and lichens), play a destructive role in their physical weakening and color changes during a biological weathering process, also known as bioerosion or biodeterioration [1,7,15,16,17,18,19,20,21,22,23].

Although bioerosion has long been recognized as a threat to the conservation of historical buildings and monuments [1,24,25,26], some evidence of the positive effects of biocolonization on inhabited surfaces has also been accumulated in recent years [27,28]. During the ongoing debate on biodeterioration vs. bioprotection [29], several authors have noted the need for the correct identification of algal inhabitants as an important step towards preventing the destruction of monuments or towards their subsequent restoration [30]. Nevertheless, documentation of their algal biodiversity is sparse [1,31]. This is particularly true for megaliths, which are traditionally defined as large, uncovered stones used in the construction of ancient structures, as sacred landmarks commemorating important places or events, or as astronomic observation sites in various civilizations [24,32,33]. The term was derived from the Greek words “mega” (great) and “lithos” (stone) and was first used regarding the Stonehenge complex in England [34]. According to the documents of the UNESCO Meeting “Megalithic Sites and the World Heritage Convention” that took place in Antequera—Málaga, Spain in 2011, “these large stones are often cut or carved, and range from single-standing stones, line or circle arrangements, simple chambers made using a lintel structure, complex buildings with multiple chambers, entrances and passages, and also appear in their natural state as part of a geological formation” [35] (p. 1). Since each megalith can be used either alone or together with other stones, there is a variety of terms depending on the number of stone blocks used and the way they are organized [36]. As there is no universally accepted conventional terminology [37], it should be noted that some of the most popular and widely accepted terms are those derived from Celtic words. These include, for example, (1) menhir (used for a high, single upright stone) which is composed of the Celtic words “mean, men” (stone) and “hir” (long, high); (2) dolmen (used for a simple, rectangular tomb covered by large vertical stone walls), which comes from the Celtic words “daul, taol, dol” (table) and “maen, men” (stone), and (3) cromlech (applied for a set of vertical stones arranged in a circular structure), also derived from the Celtic words “crom” (bent, curved) and “lech, lech” (stone) [33]. 

Various megaliths are spread all over the world, with the last estimations showing that about 35,000 of them have been discovered in Europe, representing some of the oldest cultural landscapes on the continent [32,38]. For thousands of years, they have been a significant part of the cultural landscape and today attract numerous tourists like a magnet [33]. Therefore, their distribution, function, and social significance comprise an important part of scientific research [39]. In contrast to the megaliths of Western Europe which are well documented archaeologically and enjoy great popularity [39], the megaliths of the Balkan Peninsula, are less known to the general public [40]. Some of them are recorded in north-eastern Greece and north-western Turkey, but most of these prehistoric structures are concentrated in eastern and south-eastern Bulgaria [40,41,42,43,44]. Although the petrography is not completely clarified, it is assumed that the host rocks of the Bulgarian megaliths are mainly granitoid quartz-bearing rocks (granites or gneisses) [41].

Field investigations of megaliths in Bulgaria began in the late 19th–early 20th century and continue to this day [45]. There are different opinions about the age of these ancient constructions: Most scholars date them to the Iron Age (1200–500 BC), while for some of them, the earlier Chalcolithic period (5000–3500 BC) has been hypothesized [41]. Studies have been carried out by historians, archaeologists, paleoastronomers, geographers, and geologists, but up to date, the megaliths located in Bulgaria have not been the focus of phycologists. This should be seen in the context of the numerous phycological studies which started at the end of the 19th century [46] and led to the identification of about 500 aeroterrestrial algae from soils, rocks, caves, tree barks, mosses, plants, and snow, representing 1% of the total algal biodiversity in the country [6,8,47].

This paper is the first report on the algal growth on Bulgarian megaliths. It is based on selected megaliths, situated in the Haskovo district in the southeastern part of the country. In addition to encountering algal biodiversity, the study also considers the safety aspects and points out the presence of potential toxin-producing species that could be harmful to the health of visitors to the megaliths. Last, but not least, we made a preliminary attempt to find evidence of global warming‘s influence on the algal biodiversity in such strongly exposed extremophile habitats. 

## 2. Materials and Methods

**Figure 1:**
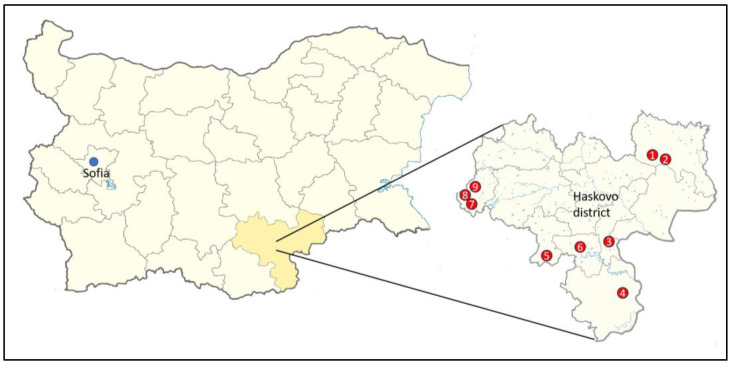
Map of Bulgaria with borders of different country districts, with enlargement of the map of the Haskovo district and indication of megalith locations: 1—Tsarski Dolmen, 2—Evdzhika, 3—Gluhite Kamuni, 4—Plevun, 5—Kovan Kaya, 6—Cromleh, 7—Angel Voyvoda, 8—Stupkata na Bogoroditsa, and 9—Sharapanite.

The materials were collected from 14–18 July 2022 from nine selected megaliths in the Haskovo district in south-eastern Bulgaria (Table 1, Figure 1). The names of the megaliths are transliterated according to the Transliteration Act of the Bulgarian Government [48].

**Figure 2:**
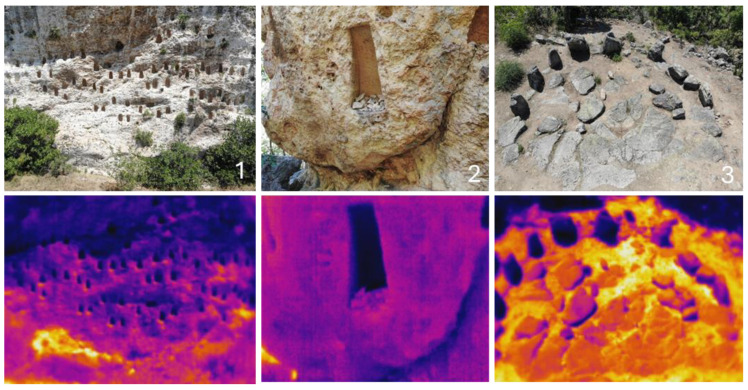
Examples of measurement of the temperature on the megalith surfaces by application of a drone: Kovan Kaya—general view (**1**) and one of the rock niches magnified (**2**), Cromlex—general view (**3**). On the lower row—the same places in the same order with differences in the temperatures measured by the drone. The temperature variations from cold to hot are expressed by blue-violet and yellow-orange colors.

Sampling sites were identified using combined land and drone observations. The megaliths were observed using a drone DJI Mavic 2 Enterprise Dual Pro (DJI Technology Co, LTD, Shenzhen, China) equipped with a photo and thermal camera (Figure 2).

From the layers with visible coloration, altogether 53 samples were scrapped following the direct collection method [49] with a medical scalpel from all available megalithic surfaces, including crevices and cracks, onto agar plates covered with Bold Basal Medium—BBM [50] (Table 1). The rock surfaces were not affected and none of the ancient monuments were damaged. To avoid destroying the megaliths, five endolithic samples were taken only from already broken pieces found in the first three megaliths. 

**Figure 3:**
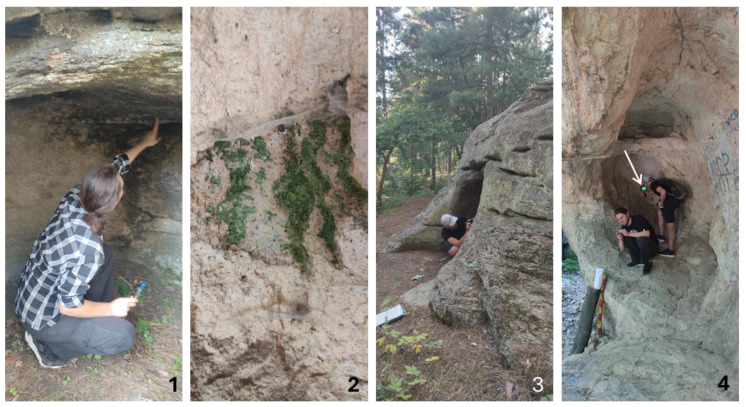
Examples of collecting the material from visible algal layers and measuring the temperature on the megalith surfaces of Evdzhika (**1**–**3**) and Gluhite Kamuni (**4**) by application of a thermal camera (indicated by white arrow).

During the collection, the temperature of the megalith surfaces (Table 1) was measured directly on each sampling site with a Bosch GTC 400C Thermo Camera which has a measurement range of −10 to +400 °C (Figure 3). Both drone and thermal cameras are applied for the first time during the sampling of aeroterrestrial algae in Bulgaria.

After collection, the Petri dishes were transported to the lab, where the material was further proceeded for future cultivation on BBM and for obtaining clone cultures by standard methods [49,50]. Each sample was investigated at regular intervals according to the visible growth of the culture. Identification of algae was performed on non-permanent slides on an Olympus BX53 light microscope. Photomicrographs were taken by an Olympus DP72 micro photo camera.

The taxonomic sources used for the determination of the algae include the standard Central European taxonomic literature (e.g., [2,3,51,52,53]) with currently published relevant papers. Since we follow the International Code for Nomenclature of Algae, Fungi, and Plants [54], the phylum name Cyanoprokaryota according to [51,52,53] is used here for the prokaryotic blue-green algae, which are also known as Cyanobacteria. All other updates of the synonymy, as well as the distribution data, follow AlgaeBase [55]. Some of the species are not yet definitely identified for objective reasons: (1) due to the absence of reproductive or resting stages, which are important diagnostic features; (2) due to the finding of some fragments in the initial samples, which did not develop during the cultivation process, or (3) due to peculiar features that do not fit the species descriptions in the available literature.

The floristic similarity was estimated according to the standard Sørensen Correlation Index [56]. The conservation status of the recorded species was checked in the Red List of Bulgarian Microalgae [57]. The potential toxin producers have been outlined following the methodology and references provided in detail in [11,12]. Considering the high surface temperatures measured (up to 49.6 °C at the most exposed places) and their broad range of about 29 °C between different sites during the quite short period of collection (five days), some correlations between the temperatures and diversity (total and by phyla) were estimated. For these estimations, the coefficient *r* from Microsoft Excel Version 2406 for Windows 11 was used and the correlation was accepted as significant if the statistical threshold value (STV) was at least *p* < 0.05 [58]. The correlations were estimated on the basis of the lowest and the highest measured temperatures for each megalith (including data on temperatures of the scrapped surfaces from which no growth was detected in the laboratory cultures) and the temperatures of each sampling point.

The terminology used follows the standard understanding of rock- and stone-associated aeroterrestrial algae as lithophytes (lithobionts), which, are further subdivided into epiliths (epilithic algae) for those inhabiting the rock surfaces and endoliths (endolithic algae) developing inside the rock substratum or in the crevices [2,3,47,59], depending on the area of colonization.

## 3. Results

A total of 90 species and 1 variety from 65 genera and 4 phyla were identified: Cyanoprokaryota (29 species from 13 genera), Chlorophyta (40 species and 1 variety from 38 genera), Streptophyta (5 species from one genus), Ochrophyta (16 species from 13 genera)—Table 2.

The number of species per sample was quite low—from 1 to 12, with 3 being the average. In 10 samples, no growth was obtained in the lab. Regarding the phyla, the average number was 1, except for Chlorophyta, for which the average number per sample was 2 species. The real number per sample in different phyla was as follows: Cyanoprokaryota (0–6), Chlorophyta (0–7), Streptophyta (0–2), and Ochrophyta (0–2). 

**Figure 4:**
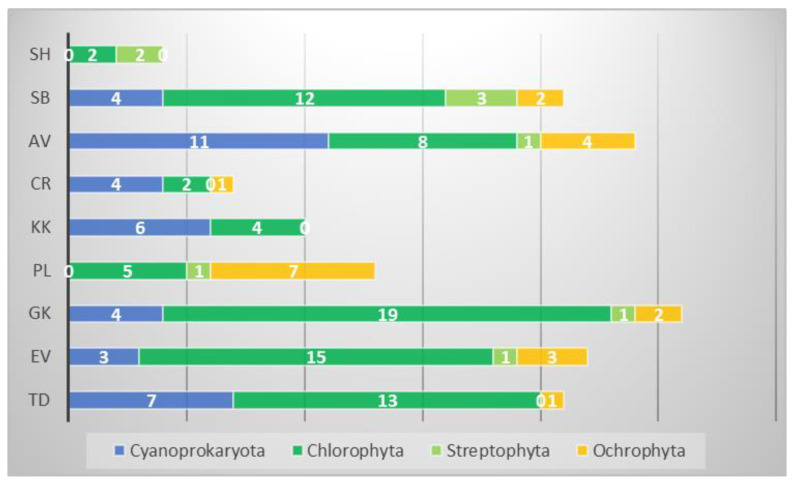
General algal biodiversity of each of the nine studied megaliths represented by the number of species in each of the main taxonomic groups (white numerals). Abbreviations on the ordinate indicate the megaliths: TD—Tsarski Dolmen, Ev—Evdzhika, GK—Gluhite Kamuni, Pl—Plevun, KK—Kovan Kaya, Cr—Cromleh, AV—Angel Voyvoda, SB—Stupkata na Bogoroditsa, Sh—Sharapanite.

The number of identified species in each of the megaliths varied between 4 and 26 (Figure 4).

**Figure 5:**
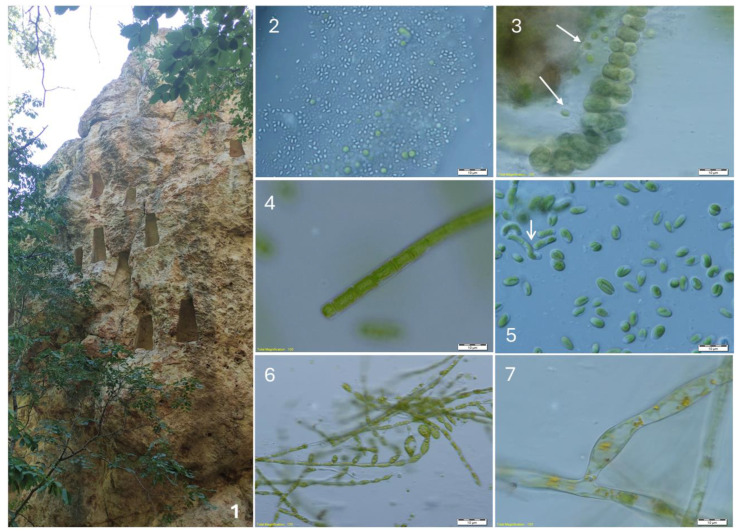
Megalithic complex Gluhite Kamuni (part of the complex with wall niches—(**1**)) and some algae growing in the obtained cultures: *Aphanothece* cf. *saxicola* (**2**), *Nostoc linckia* and *Chloroideum ellipsoideum* (arrow) (**3**), *Klebsormidium klebsii* (**4**), *Sphaerococcomyxa olivacea* and *Pseudostichococcus monallantoides* var. *exiguus* (arrow) (**5**), *Printzina lageniforme* (**6**), and *Trentepohlia* cf. *jacunda* (**7**).

The richest algal flora was found at the large Thracian cult complex Gluhite Kamuni, which included various habitats such as rock walls with specific manufactured niches (Figure 5), rock sanctuaries, and different caves. Twenty-five species and one variety were found there (Table 2, Figure 4). The most widespread in the surface layers was *Stichococcus bacillaris*, followed by *Klebsormidium klebsii*, *Pleurochloris commutata,* and *Aphanothece* cf. *saxicola* (Figure 5). *Parachlorella kessleri* and *Printzina lagenifera* were relatively common *(*Figure 5). *Trentepohlia* cf. *jucunda* was found together with *Aphanothece* cf. *saxicola* as an endolith, inside the rock surfaces (Figure 5).

**Figure 6:**
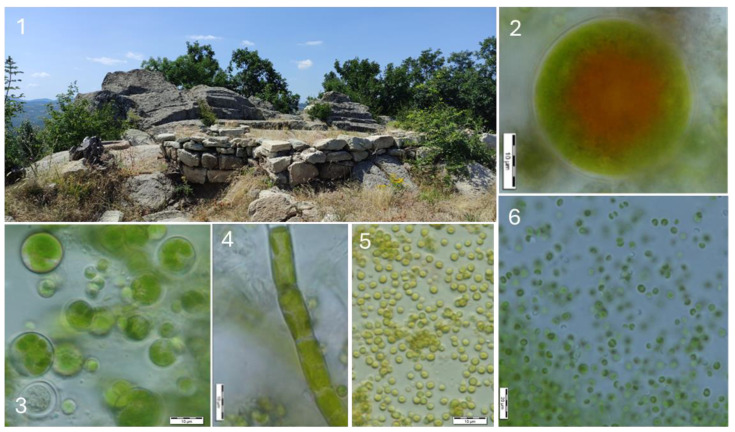
Thracian complex near village Angel Voyvoda (part of the complex with old rock sanctuaries, remnants of fortress walls, and circular rock excavations—(**1**)) with examples of some identified algae: *Pseudodictyochloris multinucleata* (**2**), *Apatococcus lobatus* (**3**), *Tribonema minus* (**4**), *Pleurochloris commutata* (**5**), and *Chloridella minuta* (**6**).

Similar vertical walls with manufactured rock niches are typical for the large Thracian cult complex Angel Voyvoda (Figure 6) from the rock surfaces of which 24 species have been identified (Table 2, Figure 4). Most of them were sparsely distributed and only *Chloroideum ellipsoideum* was found in two samples.

**Figure 7:**
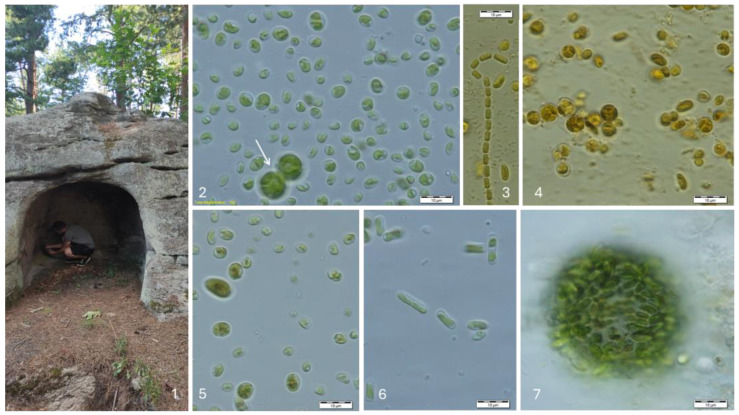
The rock sanctuary Evdzhika near the village Hlyabovo (front view—(**1**)) with examples of some species growing in the cultures: *Muriella decolor* (arrow) and *Elliptochloris bilobata* (**2**), *Stichococcus chlorelloides* (**3**), *Muriella terrestris* (**4**)*, Elliptochloris subshaerica* (**5**), *Stichococcus mirabilis* (**6**), and *Sphaerococcomyxa olivacea* (**7**). Photos (**3**–**5**) are taken after staining with Lugol’s solution.

In the Evdzhika rock sanctuary (known also as Dolmen Evdzhika), 22 species were identified (Table 2, Figure 4). The most common species was *Elliptochloris bilobata* (Figure 7), which occurred both epilithically (on the rock surfaces) and endolithically (inside the rocks). Three other species were recorded as endolithic—*Coenobotrys gloeobotrydiformis*, *Stichococcus minutus,* and *Stichococcus mirabilis* (Figure 7).

**Figure 8:**
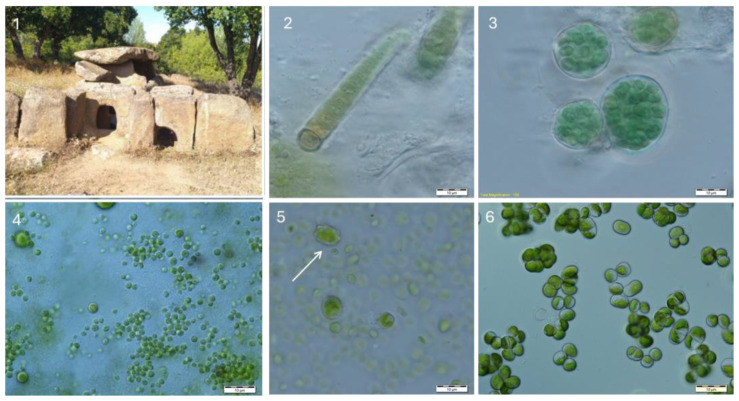
Tsarski Dolmen (**1**) and examples of species growing in the obtained cultures: *Calothrix* sp. juv. (**2**), *Nostoc minutum* (**3**), *Edaphochlorella mirabilis* (**4**), *Scotiella tuberculata* (arrow) surrounded by cells of *Chloroideum ellipsoideum* (**5**), and *Desmococcus olivaceus* (**6**).

In the two-chambered megalith Tsarski Dolmen (which in the Bulgarian language means Kings Dolmen), 21 species were identified on the rock surfaces (Table 2, Figure 4). The most common species on this megalith were *Edaphochlorella mirabilis* and *Muriella terrestris,* and, in only one sample, *Scotiella tuberculata* (Figure 8) was found.

**Figure 9:**
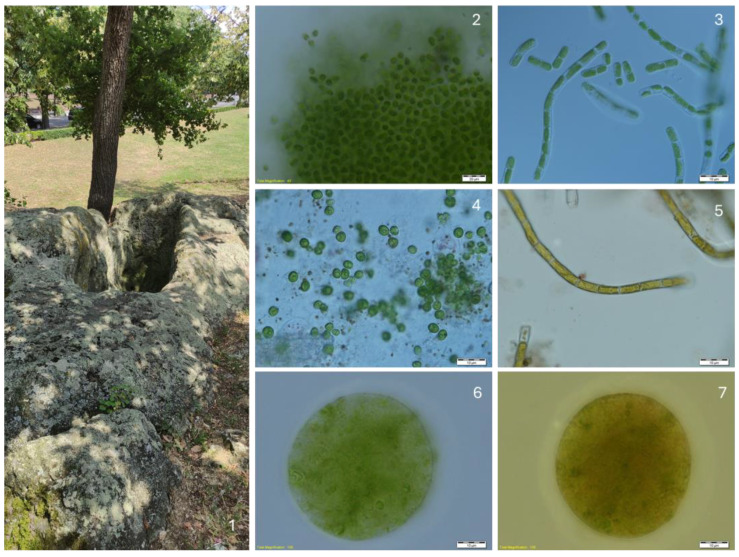
The megalith Stupkata na Bogoroditsa (**1**) and some species growing in the obtained cultures: *Choricystis parasitica* (**2**), *Klebsormidium dissectum* (**3**), *Chloroideum ellipsoideum* (**4**), *Tribonema minus* (**5**), and *Pseudodictyochloris multinucleata*—before (**6**) and after coloration by Lugol’s solution (**7**).

Twenty-one species were recorded from the megalith called Stupkata na Bogoroditsa (which means Mother Mary’s Step in Bulgarian)—Table 2, Figure 4. The most widespread species there were *Chloroideum ellipsoideum*, *Klebsormidium dissectum, Klebsormidium flaccidum*, *Pseudodictyochloris multinucleata, Stichococcus bacillaris*, and *Tribonema minus* (Figure 9).

**Figure 10:**
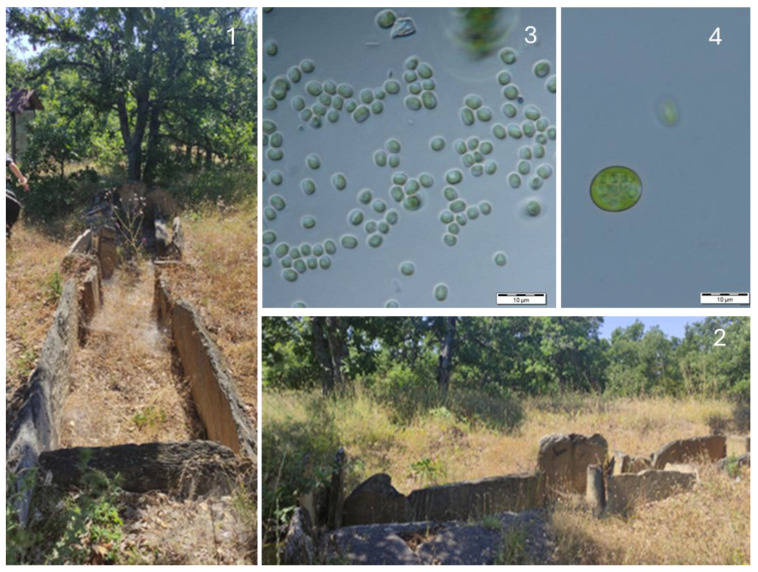
The megalith Plevun (**1**,**2**) and examples of algae from the obtained cultures: *Chloridella minuta* (**3**) and *Lobosphaeropsis lobophora* (**4**).

Thirteen species were identified on the rock surfaces of the Plevun megalith (Table 2, Figure 4 and Figure 10), none of which occurred in all sampled algal layers.

**Figure 11:**
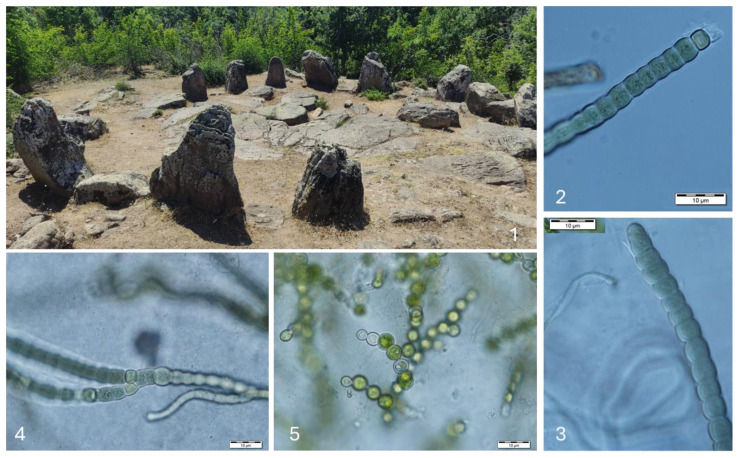
The megalith circle, Cromleh (**1**) and examples of species growing species in the collected samples: *Anabaena* sp. ster. (**2**,**3**), *Stigonema* cf. *hormoides* (**4**), and *Heterococcus anguinis* (**5**).

Seven species were detected in the megalith circle, named Cromleh (Table 2, Figure 4), the most common being *Leptolyngbya* sp. and *Anabaena* sp. ster. (Figure 11).

**Figure 12:**
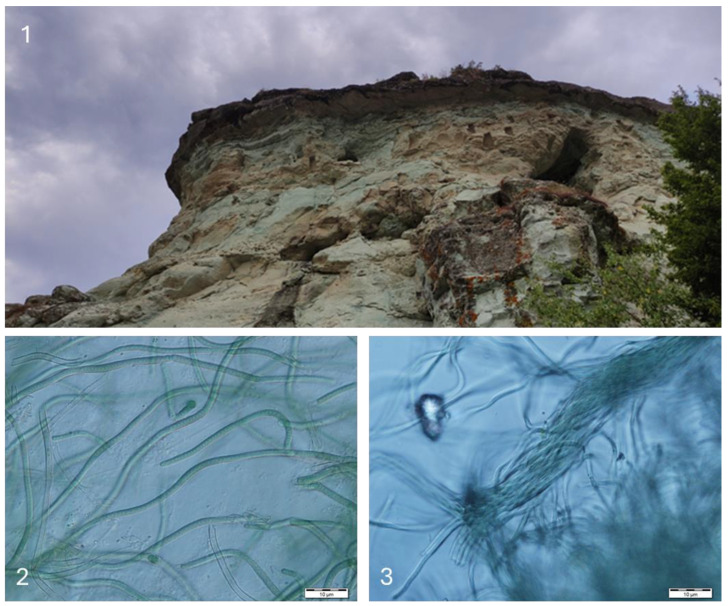
The rock niches Kovan Kaya (**1**) and examples from some species growing in the obtained cultures: *Symploca* cf. *dubia* (**2**) and *Microcoleus vaginatus* (**3**).

Ten species were identified in the samples from the Kovan Kaya complex, which contained manufactured rock niches (Table 2, Figure 4 and Figure 12). There, the only common species in the surface algal layers was *Symploca* cf. *dubia* (Figure 12).

**Figure 13:**
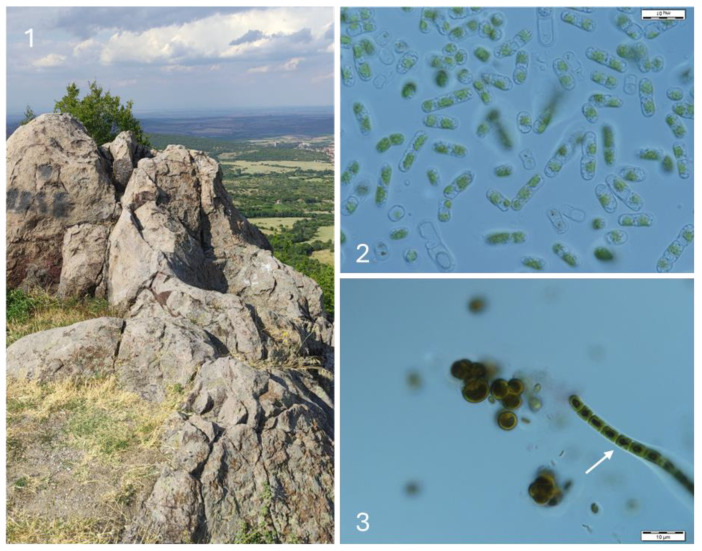
Megalith Sharapanite (**1**) and some species growing in the obtained cultures: *Klebsormidium dissectum* (**2**), *Apatococcus lobatus,* and *Klebsormidium klebsii* (arrow) after staining with Lugol’s solution (**3**).

Only four species were identified on the surfaces of the Sharapanite megalithic complex (Table 2, Figure 5 and Figure 13). There, only *Klebsormidium klebsii* (Figure 13) was found as a common species in different algal layers.

**Figure 14:**
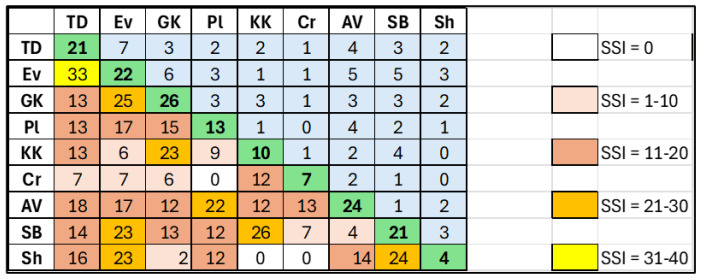
The floristic similarity between the nine studied megaliths is represented by Sørensen’s Similarity Index (SSI). The number of species in each megalith is shown with bold numerals on the diagonal (green). Above the diagonal, the number of common species for each two megaliths is shown (light blue), and below the diagonal the values of SSI are shown, grouped into five classes through 10%. Abbreviations indicate the megaliths: KD—Tsarski Dolmen, Ev—Evdzhika, GK—Gluhite Kamuni, Pl—Plevun, KK—Kovan Kaya, Cr—Cromleh, AV—Angel Voyvoda, SB—Stupkata na Bogoroditsa, Sh—Sharapanite.

The floristic similarity between the studied megaliths was very low, reaching the highest value of 33% only between the relatively closely situated Tsarski Dolmen and Evdzhika (Figure 14). The other values of SSI ranged between 0 and 25%, being between 12 and 18% in most cases (Figure 14).

**Figure 15:**
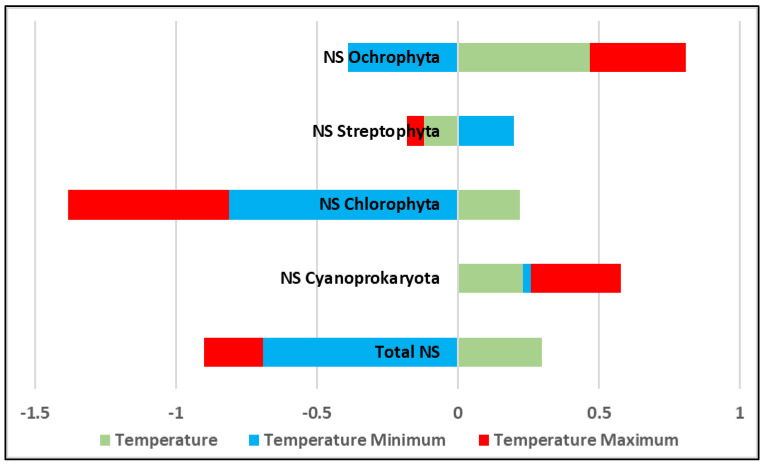
Correlations between the temperatures of the nine studied megaliths (minimum and maximum for each megalith, and by samples,) and algal biodiversity (total and by taxonomic phyla) represented by values of the correlation coefficients (when *p* < 0.05). NS—number of species.

The correlation estimated between the temperatures and total algal diversity in each sampling point was relatively low—r = 0.3 (Figure 15). Similarly, the correlations between these temperatures and separate taxonomic phyla were relatively low (Figure 15). When the generalized data for each megalith were used, the correlation between the total number of species and the lowest temperature was significant and relatively strong (r = −0.69, *p* < 0.05), whereas the correlation between the number of species and the highest measured temperature of the respected site was much lower (r = −0.21). Only Cyanoprokaryota showed positive correlations with the temperature and although the coefficient values were relatively low, it seems that the diversity of this phylum rises with increasing temperature (r = 0.33) being almost independent of the lowest temperatures (r = 0.02). A similar coefficient value was found when the number of Ochrophyta species and maximum temperature were correlated. For this group, the correlation with the minimum temperature was slightly stronger and negative (r = −0.39) (Figure 15). Negative were also the correlations between the species number of Chlorophyta and both minimum and maximum temperatures (r = −0.81 and r = −0.57, respectively). Much lower, and with different directions were the correlations estimated for the number of Streptophyta species and temperature extremes (r = 0.18 and r = −0.06, respectively).

## 4. Discussion

During the study, 90 algal species and 1 variety from 65 genera were identified (Table 2). Most of them were from the green evolutionary line, with a predominance of Chlorophyta (46% from the species diversity and 58% from the genera diversity) and significantly fewer species and genera from Streptophyta (5 and 2%, respectively)—Table 2. According to the number of species, Cyanoprokaryota outnumbered Ochrophyta (29 and 16, respectively), while the number of genera identified was similar in both phyla (13). From the large phylum Ochrophyta of the yellow-brown evolutionary line, which comprises more than 20 classes [74,75], algae from only three classes were found, namely Xanthophyceae (eight species from seven genera), Eustigmatophyceae (six species from four genera) and Bacillariophyceae (two species from two genera). The prevalence of green algae and cyanoprokaryotes supports the opinion of several authors that they are the pioneers of stone colonization (for details see [30]). The species from these two groups were the major components in most of the sampled algal layers, where they constituted two and one species on average, reaching a maximum number of species of 12 and 7, respectively.

**Figure 16:**
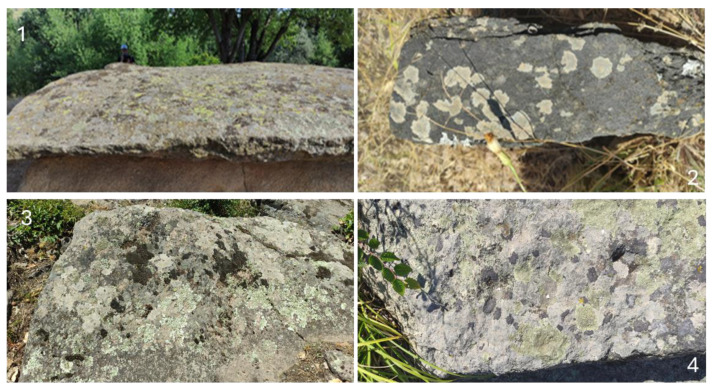
Examples of megalith surfaces covered by lichens exemplified by parts of (**1**) Tsarski Dolmen; (**2**) Megalith Plevun; (**3**) Cromleh; and (**4**) Angel Voyvoda.

**Figure 17:**
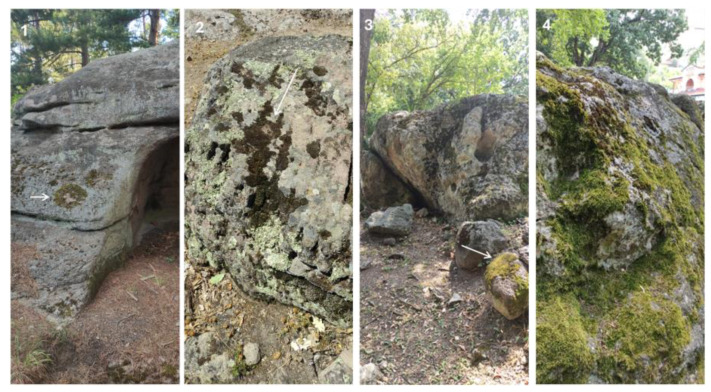
Megalith surfaces with mosses (arrows) exemplified by parts of (**1**) Evdzhika; (**2**) Cromleh; (**3,4**) and Stupkata na Bogoroditsa.

Although there are numerous publications on the algae of historical buildings and monuments [1], few studies are concerned with the algal growth on megaliths or other monuments utilizing natural rocks in different parts of the world. Moreover, they have all been obtained using different methods and concern different taxonomic categories of algae [7,76,77,78,79,80]. Some studies have focused on the soils in which megaliths were submerged (e.g., [10]). Therefore, the comparisons with all these data are extremely cautious. In general, the published algal flora from various megaliths is not rich and abundant in contrast to the mass growth of lichens and some mosses that have developed to varying degrees on the megalith surfaces [76,77,80]. At this point, reference should, therefore, be made to the interesting results of studies from the Netherlands, which show that terrestrial arthropods feed on epilithic algae, thus leaving more space for lichens [77]. In agreement with these observations, all megaliths located in open areas in the Haskovo district were largely covered by lichens (Figure 16), while algae and mosses occupied a much smaller part of their surface. The latter group was better developed only on some stones from the Stupkata na Bogoroditsa complex (Figure 17).

**Figure 18:**
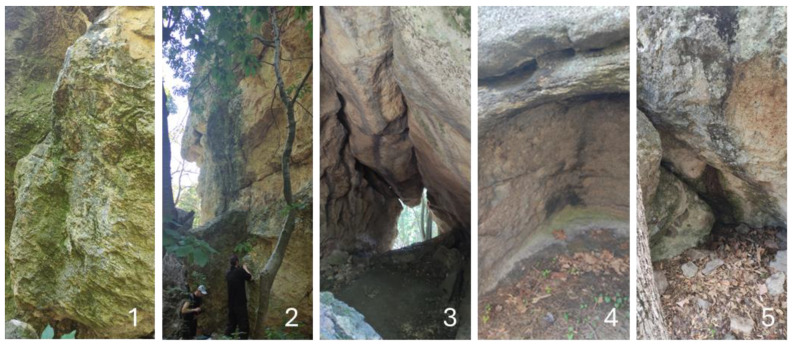
Examples of megalith surfaces with visible algal layers: (**1,2**) different parts of the Gluhite Kamuni with visible green layers on the outer surfaces of the sandy rocks in the deciduous forest; (**3**) Open rock pass Utrobata (which in the Bulgarian language means Uterus) in the complex Gluhite Kamuni with greenish layers in the inner parts; (**4**) inner part of the Evdzhika with well-developed green layer; (**5**) inner part of Stupkata na Bogoroditsa with visible green algal layers.

The algal layers were better developed on the shadier surfaces of the megaliths located in forest habitats or in the inner surfaces of the megaliths (Figure 18).

The species composition obtained during this study, is generally consistent with: (1) the culture-dependent investigations of the phototrophic community from the limestones of the World Heritage site the ‘University of Coimbra—*Alta* and *Sofia*’ (Portugal) [7], which identified green microalgae from the clades *Prasiolales*, *Chlorellales*, *Watanabea*, *Chlamydomonadales*, and *Sphaeropleales* and cyanoprokaryotes from the clades Nostocales and Synechococcales; (2) summarized data on the cyanoprokaryotes and green algae that have been identified on the European historical monuments in the Mediterranean formed by marble, limestone, travertine, dolomite, sandstone and granite, among which were the paleolithic sculptures in Angles-sur-l’Anglin [30,81]; (3) culture-dependent and culture-independent approaches in the study of the endolithic algae from the exposed dolomites in the alpine Piora Valley (Switzerland), which demonstrated the presence of the chlorophytes *Chlorella sorokiniana* and *Stichococcus bacillaris,* and of the cyanoprokaryotes *Calothrix, Chroococcidiopsis, Leptolyngbya, Microcoleus*, *Nostoc, Scytonema,* and *Symploca* [82].

As this is the first study on the algal biodiversity on megaliths in Bulgaria, considering the aerophytic mode of life on rocks, it is possible to compare the currently obtained results with those from previous studies on lithophytic and other aeroterrestrial algae in the country, based on cultivation methods [31,49,50,51,52,53,54,55,56,57,58,59,60,61,62,63,64,65,66,67,68,69,70,71,72,73]. Although the main taxonomic groups and genera are similar, the main difference lies in the much higher number of taxa identified from the megaliths (91 algae) compared to those on the epilithic algae from historical monuments, open caves, and Belogradchik rocks [31,63,66,69,70,73]. In this way, 48 identified algae are reported for the first time in the country as lithophytic: 10 cyanoprokaryotes, 19 chlorophytes, and 19 ochrophytes (Table 2). Most of them, with the exception of *Pinnularia* sp., *Tribonema aequale,* and *Tribonema minus*, are new records for the country. Only six algae were found to grow endolithically: *Aphanothece* cf. *saxicola*, *Coenobotrys gloeobotrydiformis*, *Elliptochloris bilobata*, *Stichococcus minutus*, *Stichococcus mirabilis*, and *Trentepohlia* cf. *jucunda*. In our previous studies of the Belogradchik rocks [69], endolithic development was demonstrated for 11 algae, among which were *Stichococcus* sp. and *Trentepohlia* sp. Subsequent detailed morphological analysis of the cultural material for *Stichococcus* revealed its similarity to *Stichococcus bacillaris* var. *minor* [63,66], which was considered an uncertain taxon at the time of the study [2] but is currently considered a synonym of *Stichococcus bacillaris* [3,55]. The presence and the interpenetration of *Trentepohlia* between sandstone grains to an average depth of 0.26 mm has been demonstrated in the ruins of Angkor temples in Cambodia [29]. Species of *Trentepohlia* and *Stichococcus*, as well as of *Chlorella* and *Klebsormidium*, were observed as endolithic on churches in Portugal and Spain [30,83].

The study revealed differences in the algal flora of individual megaliths, with no common species for all of them. This is reflected by the low floristic similarity between the sites (Figure 14). The greatest biodiversity was found in the large Thracian cult complex Gluhite Kamuni (25), followed by the single megalith Evdzhika (22), while the lowest number of species was found on the megalithic complex Sharapanite (4)—Table 2, Figure 4. Consistent with the overall biodiversity recorded, and with the average number of species per sample, chlorophyte species prevailed on all megaliths, except Cromleh, where cyanoprokaryotes were the richest taxonomic group. In contrast, not a single cyanoprokaryote was found on the Sharapanite megalithic complex. Only green algae from two genera, *Stichococcus* and *Klebsormidium*, were found there. This evidence, together with the finding of various *Klebsormidium* species on five other megaliths, is consistent with data on the strong surveillance abilities of algae of this genus, which can withstand drought, temperature extremes, strong insolation, and can grow in a wide pH range (e.g., [1,71,84,85,86,87,88]). *Stichococcus* is common on stone monuments of temperate and tropical regions [30,83,89]. *Stichococcus bacillaris,* in particular, was one of the most widespread algae, found in the surface layers of eight of the nine studied megaliths (Table 1). These results agree with the ubiquitous cosmopolitan distribution of this alga [2,3,55] which has been recorded from aeroterrestrial habitats in Bulgaria [47,60]. The other two widespread algae were the chlorophytes *Apatococcus lobatus* and *Chloroidium ellipsoideum*, which were found on five and four megaliths, respectively (Table 2). *Chloroidium* has a broader distribution, recorded in Europe, Asia, South America, Australia, and New Zealand [55], while the number of records of *Apatococcus lobatus* is lower and originates in Europe, Asia, Australia, and New Zealand [55]. However, *Apatococcus* has often been found to be dominant among aerophytic green algal communities due to its highly competitive strength and strong resistance combination of adaptive traits on morphological, ecophysiological, and biochemical levels [90]. These characteristics allow it to live in overshadowed and moist microclimatic conditions, but also in towns with air pollution and relatively drier air conditions (e.g., [31,91]).

In view of the relatively small number of megaliths studied, it is difficult to make a clear statement about the relationship between diversity and rock substrates, or megalith area. At first glance, however, it can be concluded that diversity was greater in the shadier and relatively colder places, which most likely explains the lowest number of species in the most exposed complexes of Cromleh and Sharapanite. This could be related to the pilot data obtained on the relatively strong significant correlation between total algal biodiversity and minimum temperature contrasted with the much lower correlation with the highest measured temperature (Figure 15). Considering the low total number of species in each algal layer (three on average) and the low correlations between temperatures and the number of species in certain phyla, estimated by samples (Figure 15), it is difficult to point to one phylum as a good indicator regarding temperature changes. However, some trends for increasing species number with rising temperatures could be predicted for cyanoprokaryotes and ochrophytes according to the low, but positive correlations estimated regarding the maximum temperatures (Figure 15). The surprising, at first glimpse, negative correlations of Chlorophyta with temperature extremes, most probably, could be explained by the autecology of the recorded species and by the fact that the highest constant temperature at which green algae were reliably identified as growing in Bulgarian thermal springs was 41 °C [91,92], while the maximum temperature measured by us was 49.6 °C (Table 1). The finding of certain (mainly chlorophyte and cyanoprokaryote) species on the most exposed surfaces and estimations of some stronger correlations between species diversity of certain phyla and temperature extremes (minimum and maximum measured values), shows the perspective of further detailed analysis based on the autecology of the species (which was beyond the scope of this study) and could stimulate future studies based on more samplings from different megaliths. Such investigations seem to be more necessary considering one of the greatest modern challenges that affects historical monuments, namely climate change [77]. Although, for more than two decades, it has been shown that global warming promotes the growth of aeroterrestrial algae [1], studies of its impact on archaeological monuments and other aeroterrestrial environments are limited [1,77].

Some of the species recorded were of conservation importance. One of these was *Pseudodictyochloris multinucleata*, which is considered extremely rare on a global scale. To date, according to AlgaeBase [55] and the comprehensive *Syllabus der Boden-, Luft- und Flechtenalgen* [2,3], it has been reported in five publications [93,94,95,96,97], mainly in the cold deserts of the Arctic, Antarctic, and alpine regions of the world. In Bulgaria, the species was previously found only once in alpine soils from Pirin Mts [60,66,67] and was included in the Red List of microalgae under the category “Endangered” [57]. Therefore, the two recent finds in much lower and warmer localities, such as Angel Voyvoda and Stupkata na Bogoroditsa megaliths, are of particular importance.

Another species of interest is *Scotiella tuberculata*, recorded for the first time in Bulgaria and, in our opinion, needs to be included in the current update of the Red List of Bulgarian Microalgae [98]. In addition, this species has rarely been documented on a global scale [2,3,67], with a few findings in Europe [99,100] and India [101].

We found one more endangered species from the Red List of Bulgarian Microalgae, namely *Vischeria stellata* [57].

As some aeroterrestrial algae produce toxins, the species from the genera *Anabaena* s.l., *Aphanocapsa*, *Aphanothece*, *Calothrix*, *Leptolyngbya*, *Microcoleus*, *Nostoc, Schizothrix,* and *Scytonema* can be potential sources of cyanotoxins harmful for the health of visitors to megaliths [11,12].

## 5. Conclusions

The present study demonstrates the rich biodiversity of prokaryotic and eukaryotic algae that live attached to the nine selected megaliths in Bulgaria, with green algae predominating in the analyzed layers. The finding of rare and threatened species and the low similarity between different rock complexes clearly show the need for further research on the numerous other megaliths scattered around the country. The algal flora varied significantly from site to site, showing low floristic similarity (0–33%) between the sites and there were no common species found on all megaliths. However, the results obtained so far suggest that the algal diversity was not primarily related to the area and substrate of the megaliths, but mainly to the environmental factors. The obtained data on correlations between the number of species, total and in specific taxonomic groups recorded at each site and the temperature could stimulate further studies based on autecological data regarding the response of the lithophytes to global warming. A final concern relates to the safety and health of tourists, as algae from nine of the found genera have been reported as potential toxin producers.

## Figures and Tables

**Table 1:** The location of the selected megaliths from the Haskovo district, represented by geographical coordinates, temperature range of the surfaces from which samples have been collected, and number of collected samples (NCS).

Megalith	Geographic Coordinates	Temperature Range [°C]	NCS
Tsarski Dolmen (=Dolmen Nachovi Chairi)	42.051401, 26.228585	25.0–33.5	5
Evdzhika	42.046864, 26.261856	19.8–26.4	5
Ancient complex Gluhite Kamuni	41.727805, 25.955231	18.1–27.2	15
Plevun	41.481050, 26.004420	25.0–42.0	3
Kovan Kaya	41.617610, 25.722950	31.1–32.2	2
Cromleh	41.681972, 25.812750	32.0–47.7	2
Ancient complex Angel Voyvoda	41.833155, 25.262709	28.4–49.6	9
Stupkata na Bogoroditsa	41.940107, 25.345984	31.5–37.9	9
Sharapanite	41.919580, 25.316216	32.8–33.5	2

**Table 2:** Species composition of the investigated megaliths from Haskovo district, South-Eastern Bulgaria: TD—Tsarski Dolmen, Ev—Evdzhika, GK—Gluhite Kamuni, Pl—Plevun, KK—Kovan Kaya, Cr—Cromleh, AV—Angel Voyvoda, SB—Stupkata na Bogoroditsa, and Sh—Sharapanite. Inside the phyla, species are organized in alphabetical order. Previous records of each species in the aeroterrestrial flora of Bulgaria (PRAFB) are indicated by relevant references [31,47,48,49,50,51,52,53,54,55,56,57,58,59,60,61,62,63,64,65,66,67,68,69,70,71,72,73].

Species/Megaliths	TD	Ev	GK	Pl	KK	Cr	AV	SB	Sh	PRAFB
CYANOPROKARYOTA										
*Anabaena* sp. ster. 1 (?*Trichormus* sp.)	x									
*Anabaena* sp. ster. 2 (?*Isocystis* sp.)		x								
*Anabaena* sp. ster. 3						x				[47]
*Aphanocapsa fusco-lutea* Hansgirg			x							
*Aphanocapsa* cf. *rivularis* (Carmichael) Rabenhorst						x				
*Aphanocapsa* sp. 1		x					x			
*Aphanocapsa* sp. 2			x				x			
*Aphanothece* cf. *saxicola* Nägeli			x							[47,60]
*Aphanothece* sp. 1	x									
*Aphanothece* sp. 2		x			x		x			
*Calothrix* sp. juv. (ad *Calothrix fusca* Bornet and Flahault)	x									
*Gloeobacter violaceus* Rippka, J. B. Waterbury and Cohen-Bazire					x		x			
*Gloeothece confluens* Nägeli							x			
*Leptolyngbya foveolarum* (Gomont) Anagnostidis and Komárek								x		[47]
*Leptolyngbya* af. *gloeophila* (Borzì) Anagnostidis and Komárek							x			
*Leptolyngbya* cf. *subtilissima* (Hansgirg) Komárek							x			
*Leptolyngbya* ‘Albertano-Kovacik green’ 1992							x			
*Leptolyngbya* sp. 1 (ad *Leptolyngbya compacta* Komárek)					x					
*Leptolyngbya* sp. 2								x		
*Leptolyngbya* sp. 3							x			
*Leptolyngbya* sp. 4 (? *Leiblenia* sp.)						x				
*Microcoleus vaginatus* Gomont					x					[47]
*Nostoc linckia* Bornet ex Bornet and Flahault	x		x		x					[47]
*Nostoc minutum* Desmazières ex Bornet and Flahault	x									
*Pseudophormidium hollerbachianum* (Elenkin) Anagnostidis							x			[60]
*Schizothrix* cf. *epilithica* (Ercegović) Anagnostidis								x		
*Scytonema* sp.	x									[47]
*Stigonema* cf. *hormoides* Bornet and Fhault	x					x	x			[47]
*Symploca* cf. *dubia Gomont* (?*Leptolyngbya* sp.)					x			x		
CHLOROPHYTA										
*Apatococcus lobatus* (Chodat) Petersen	x	x					x	x	x	[31,61,62,63]
*Chlorella vulgaris* Beijerinck								x		[47,60,62,63]
*Chlorella* sp.			x							
*Chloroidium ellipsoideum* (Gerneck) Darienko and al.	x	x	x				x	x		[47,48,49,50,51,52,53,54,55,56,57,58,59,60,61,63]
*Choricystis parasitica* (Brandt) Pröschold and Darienko		x		x				x		[60]
*Chromochloris zofingiensis* (Dönz) Fucíková and Lewis							x			[63]
*Coccomyxa subglobosa* Pascher			x		x			x		
*Coelastrella terrestris* (Reisigl) Hegewald and Hanagata			x	x						[60,64]
*Coenobotrys gloeobotrydiformis* (Reisigl) Kostikov and al.		x								[60,63]
*Desmococcus olivaceus* (Persoon ex Acharius) Laundon	x									[61,62,63,65]
*Deuterostichococcus tetrallantoideus* (Kol) Pröschold and Darienko			x							
*Edaphochlorella mirabilis* (Andreeva) Darienko and Pröschold	**x**		x							[63]
*Elliptochloris bilobata* Tschermak-Woess		x								[60]
*Elliptochloris subsphaerica* (Reisigl) Ettl and Gärtner		x	x							[63]
*Eubrownia aggregata* (R. M. Brown and Bold) Shin Watanabe and Lewis					x					
*Hemichloris polyspora* Tschermak-Woess, Hua, Gärtner and Hesse								x		
*Lobosphaera undulata* (Shin Watanabe) Ettl and Gärtner	x									
*Lobosphaeropsis lobophora* (Andreeva) Ettl and Gärtner	x	x		x						[63]
*Monoraphidium nanum* (Ettl) Hindák		x								
*Muriella decolor* Vischer		x								
*Muriella terrestris* J. B. Petersen	x	x								
*Mychonastes homosphaera* (Skuja) Kalina and Puncochárová	x						x			[61,62,63,66,68]
*Neocystis brevis* (Vischer) Kostikov and Hoffmann			x							
*Parachlorella kessleri* (Fott and Nováková) Krienitz and al.			x							
*Pleurastrum minutum* (Starr) Sciuto and al.					x	x		x		
*Printzina lagenifera* (Hildebrand) Thompson and Wujek			x							
*Pseudodictyochloris multinucleata* (Broady) Ettl and Gärtner							x	x		[60,66,67]
*Pseudostichococcus monallantoides* var. *exiguus* (Gerneck) Pröschold and Darienko				x						[63]
*Radiococcus bilobatus* (Broady) Kostikov and al.			x							
*Scotiella tuberculata* Bourrelly	x									
*Sphaerococcomyxa olivacea* (Petersen) Kostikov and al.		x	x					x		
*Stichococcus bacillaris* Nägeli	x	x	x	x	x		x	x	x	[47,60,62,63,65,66,69]
*Stichococcus chlorelloides* Grintzesco and Ș. Péterfi		x								
*Stichococcus minutus* Grintzesco and Ș. Péterfi	x	x								[60,63,65]
*Stichococcus mirabilis* Lagerheim		x	x					x		
*Tetracystis pulchra* R. M. Brown and Bold				x			x			
*Trebouxia* sp. (*ad Asterochloris excentrica* (Archibald) Skaloud and Peksa)			x							[47,61]
*Trentepohlia arborum* (Agardh) Hariot			x			x	x			
*Trentepohlia jolithus* (Linnaeus) Wallroth			x							[47]
*Trentepohlia* cf. *jucunda* (Cesati) Hariot			x							
*Uvulifera mucosa* (Broady and Ingerfeld) Molinari	x									
STREPTOPHYTA										
*Klebsormidium crenulatum* (Kützing) Lokhorst								x		
*Klebsormidium dissectum* (Gay) Ettl and Gärtner								x	x	[60,62,70,71]
*Klebsormidium flaccidum* (Kützing) Silva, Mattox and Blackwell							x	x		[47,60,63,71]
*Klebsormidium* cf. *flaccidum* (Kützing) Silva, Mattox, and Blackwell				x						
*Klebsormidium klebsii* (Smith) Silva, Mattox and Blackwell		x	x						x	[63,69]
OCHROPHYTA										
*Botrydiopsis* sp.				x						
*Chlorellidium astigmatum* Schwarz							x			
*Chloridella minuta* Gayral and Mazancourt				x			x			
*Chlorobotrys gloeothece* Pascher		x								
*Ellipsoidion perminimum* Pascher	x			x						
*Gloeobotrys piriformis* Reisigl				x						
*Gloeobotrys terrestris* Reisigl				x						
*Heterococcus anguinus* Pitschmann						x				
*Monodus guttula* Pascher		x								
*Navicula* sp. s.l.							x			[47]
*Pinnularia* sp.		x								
*Pleurochloris commutata* Pascher			x	x			x			
*Tribonema aequale* Pascher								x		
*Tribonema minus* (Wille) Hazen								x		
*Vischeria magna* (Petersen) Kryvenda and al.			x							[60,63,66,72]
*Vischeria stellata* (Chodat) Pascher				x						[60,63,66,69,72,73]

## Data Availability

Data contained within the article.
